# Did You Know That Confounding Could Bias Results Obtained within an Economic Evaluation?

**DOI:** 10.1177/0272989X261474826

**Published:** 2026-09-04

**Authors:** Simon LaRue, Mike Paulden, Denis Talbot, Jason Robert Guertin

**Affiliations:** 1 Axe santé des populations et pratiques optimales en santé, Centre de recherche du CHU de Québec-Université Laval, Quebec City, Canada; 2 School of Public Health, University of Alberta, Edmonton, Canada; 3 Département de médecine sociale et préventive, Université Laval, Quebec City, Canada; 4 Centre de recherche en organogénèse expérimentale de l’Université Laval/LOEX, Quebec City, Canada

**Keywords:** economic evaluation, observational studies, confounding bias, real-world evidence, real-world data

## Abstract

**Background:**

The increasing use of real-world data in economic evaluation raises concerns about the potential for confounding bias. Methods to control for this bias have been developed, but there is still much to be understood about how confounding influences economic evaluations.

**Objectives:**

To illustrate the impact of unadjusted confounding variables in economic evaluations of observational studies.

**Methods:**

We simulated the costs and effectiveness of 2 treatments across 9 possible confounding effect scenarios. We considered these scenarios in the context in which one treatment is more costly and more effective with mild correlation between confounders and outcomes. All scenarios examined the incremental cost and effectiveness of 400 randomly generated individuals, reflecting sample sizes commonly seen within observational economic evaluations. Results were illustrated with the use of cost-effectiveness planes and cost-effectiveness acceptability curves (CEAC).

**Results:**

Our simulations illustrate that confounding bias can have a significant effect on incremental costs and incremental effectiveness estimates. These simulations also illustrate that confounders can affect the evaluation of uncertainty by causing a shift in the CEACs. Such results hint that inadequate consideration of confounding bias can potentially lead to flawed judgments about the cost-effectiveness of a treatment.

**Discussion:**

Results of economic evaluations can be influenced by confounding when they are conducted in an observational setting. Efforts must be made to limit their impact to ensure an accurate assessment of the economic value of treatments and to prevent potential losses of population health.

**Highlights**

- Eight different types of confounding effects can affects the results obtained within an observational economic evaluation.
- Although all types of confounding effects will bias the results, their effect on the conclusion of the cost-effectiveness profile of treatment over another can substantially differ.

## Introduction

An economic evaluation of a health technology is an interdisciplinary assessment that combines clinical and epidemiological aspects of a treatment with its economic consequences. This line of work seeks to employ evidence-based data to evaluate the benefits of a health program in relation to the resources it consumes.^1,2^ However, integrating multiple aspects of each discipline into a single randomized controlled trial may be challenging due to limitations in cost and sampling capacity.^3^ To circumvent these limitations, observational studies (also referred to as nonrandomized studies) can serve as valuable sources of information, as they can capture real-world data of cost and effectiveness on a targeted population and are often more feasible than a randomized control trial.^4,5^ Despite their strengths, observational studies may also introduce confounding bias into the data, as patients are not randomly assigned to treatment groups. Failure to adjust for this bias within an observational economic evaluation may result in an incorrect assessment of the economic value of the different treatments.^6–8^

Although several methods have been proposed to adjust for this bias within observational economic evaluations^9^ (eg, propensity scores,^10^ seemingly unrelated regression,^11^ net monetary benefit [NMB] regression,^12^ covariate matching,^13^ and Bayesian inference^14^), many groups still fail to adjust for confounding bias when conducting such studies.^15–17^ We therefore aimed to highlight the impact of confounders in observational studies and the different effects they can have on the results of an economic evaluation through a series of scenarios based on synthetic data.

## Method

### Simulation Design

To consider the influence of uncontrolled confounders on economic evaluation studies, we simulated a 2-arm observational trial. The use of a simulated database enabled us to examine specific instances in which confounding is present, either on a single outcome of cost or effectiveness, or on both outcomes simultaneously.

We first created a scenario depicting a context in which there is no confounding effect on both cost and effectiveness (ie, an unbiased setting; hereby identified as scenario V within our simulations). We then generated 8 additional scenarios, each demonstrating how a linear confounding effect could affect the simulated data in 1 of the 8 radial directions of the cost-effectiveness (CE) plane:

I. An increase in the estimated costs and a decrease in the estimated effectiveness
II. An increase in only the estimated costs
III. An increase in both the estimated costs and effectiveness
IV. A decrease in only the estimated effectiveness
V. No change in estimated costs and effectiveness
VI. An increase in only the estimated effectiveness
VII. A decrease in both the estimated costs and effectiveness
VIII. A decrease in only the estimated costs
IX. A decrease in the estimated costs and an increase in the estimated effectiveness

We used scenario V as the reference when comparing results obtained with the other 8 confounding effects. In short, the 9 scenarios proposed correspond to the 9 possible situations that could arise in an economic evaluation conducted in an observational setting.

For each of these 9 scenarios, we considered a situation in which a new treatment is both more costly and more effective than the comparator, with a true incremental cost-effectiveness ratio (ICER) exactly equal to the willingness-to-pay threshold (in this case $50,000 per quality-adjusted life-year [QALY]). Under such a setting, the new treatment should be as likely to be considered cost-effective (50%) as not cost-effective (50%).

### Data-Generating Process

For each scenario, we generated 8 independent covariates $X_{j},j=1,\ldots ,8$ , a binary treatment variable t , a cost outcome variable c , and an effectiveness outcome variable e for 400 individuals. The covariates were generated independently according to a normal distribution of mean 0 and standard deviation of 1. The treatment variable was generated according to a Bernoulli distribution with $P(t=1|X)=\mathrm{expit}(0.3+0.4\Sigma_{j}X_{j})$ , where expit is the inverse of the logit function. This produces approximately 56% of observations in the treatment group and standardized mean differences between treatment and control groups of approximately 0.32 (95% confidence interval [CI] 0.122–0.522) for each of the 8 covariates. Costs were generated according to a Gamma distribution. The parameters of the Gamma distribution were chosen such that the standard deviation was $12,500 and the mean was $\$50,000+\$5,000t+\beta_{2}^{c}X_{1}$ , with the value of $\beta_{2}^{c}$ varying between scenarios to induce a different direction of confounding. Effectiveness was generated according to a normal distribution with a standard deviation of 0.2 and a mean of $0.8+0.1t+\beta_{2}^{e}X_{1}$ , where the value of $\beta_{2}^{e}$ again varied between scenarios. In scenarios in which the covariate $X_{1}$ affected costs or effectiveness, or both, the coefficients $\beta_{2}^{c}$ and $\beta_{2}^{e}$ were chosen such that the correlation between the covariate $X_{1}$ and both cost and effectiveness was approximately ±0.10 conditional on treatment; $\beta_{2}^{c}$ and $\beta_{2}^{e}$ were fixed to zero otherwise. More details on the generation of costs and on the choice of $\beta_{2}^{c}$ and $\beta_{2}^{e}$ are provided in the appendix.

Table 1 details how confounding effects were included within the 9 scenarios. $\beta_{2,k}^{c}$ and $\beta_{2,k}^{e}$ were set to 0 in the reference scenario (V) to reflect the absence of confounding.

**Table 1.** Confounding Effect on Coefficient Values.

Scenario	Cost Coefficient	Effectiveness Coefficient
I	$\frac{\sigma_{c}}{\sigma_{X}}\sqrt{\frac{r_{c}^{2}}{1+r_{c}^{2}}}$	$-\frac{\sigma_{e}}{\sigma_{X}}\sqrt{\frac{r_{e}^{2}}{1+r_{e}^{2}}}$
II	$\frac{\sigma_{c}}{\sigma_{X}}\sqrt{\frac{r_{c}^{2}}{1+r_{c}^{2}}}$	0
III	$\frac{\sigma_{c}}{\sigma_{X}}\sqrt{\frac{r_{c}^{2}}{1+r_{c}^{2}}}$	$\frac{\sigma_{e}}{\sigma_{X}}\sqrt{\frac{r_{e}^{2}}{1+r_{e}^{2}}}$
IV	0	$-\frac{\sigma_{e}}{\sigma_{X}}\sqrt{\frac{r_{e}^{2}}{1+r_{e}^{2}}}$
V	0	0
VI	0	$\frac{\sigma_{e}}{\sigma_{X}}\sqrt{\frac{r_{e}^{2}}{\left(1+r_{e}^{2}\right)}}$
VII	$-\frac{\sigma_{c}}{\sigma_{X}}\sqrt{\frac{r_{c}^{2}}{1+r_{c}^{2}}}$	$-\frac{\sigma_{e}}{\sigma_{X}}\sqrt{\frac{r_{e}^{2}}{1+r_{e}^{2}}}$
VIII	$-\frac{\sigma_{c}}{\sigma_{X}}\sqrt{\frac{r_{c}^{2}}{1+r_{c}^{2}}}$	0
IX	$-\frac{\sigma_{c}}{\sigma_{X}}\sqrt{\frac{r_{c}^{2}}{1+r_{c}^{2}}}$	$\frac{\sigma_{e}}{\sigma_{X}}\sqrt{\frac{r_{e}^{2}}{1+r_{e}^{2}}}$

From our simulated dataset, we calculated the unadjusted incremental costs, unadjusted incremental effectiveness, and the unadjusted ICERs in each scenario. We repeated the data-generation procedure 10,000 times for each scenario. Data simulation and analyses were performed using R 4.5.1 software.^18^

### Analysis

To examine the influence of confounders on the data, we generated a panel of 9 scatterplots of incremental costs and incremental effectiveness on the CE plane, 1 for each scenario. The proportion of points in each quadrant of the CE plane was also calculated for each scenario.

We also considered how the influence of confounders could affect the results of the CE analyses (ie, which intervention is the most cost-effective and the probability that each intervention is cost-effective). This secondary analysis is important as a change in the ICER estimate or the incremental NMB does not necessarily result in a change in this conclusion. To consider which intervention is cost-effective, we plotted the cost-effectiveness acceptability curves (CEACs), which reflect the proportion of iterations in which each intervention has the highest expected NMB, given a specific threshold value. The curves were produced using threshold values ranging from $0 to $150,000 per QALY.

## Results

**Figure 1.**
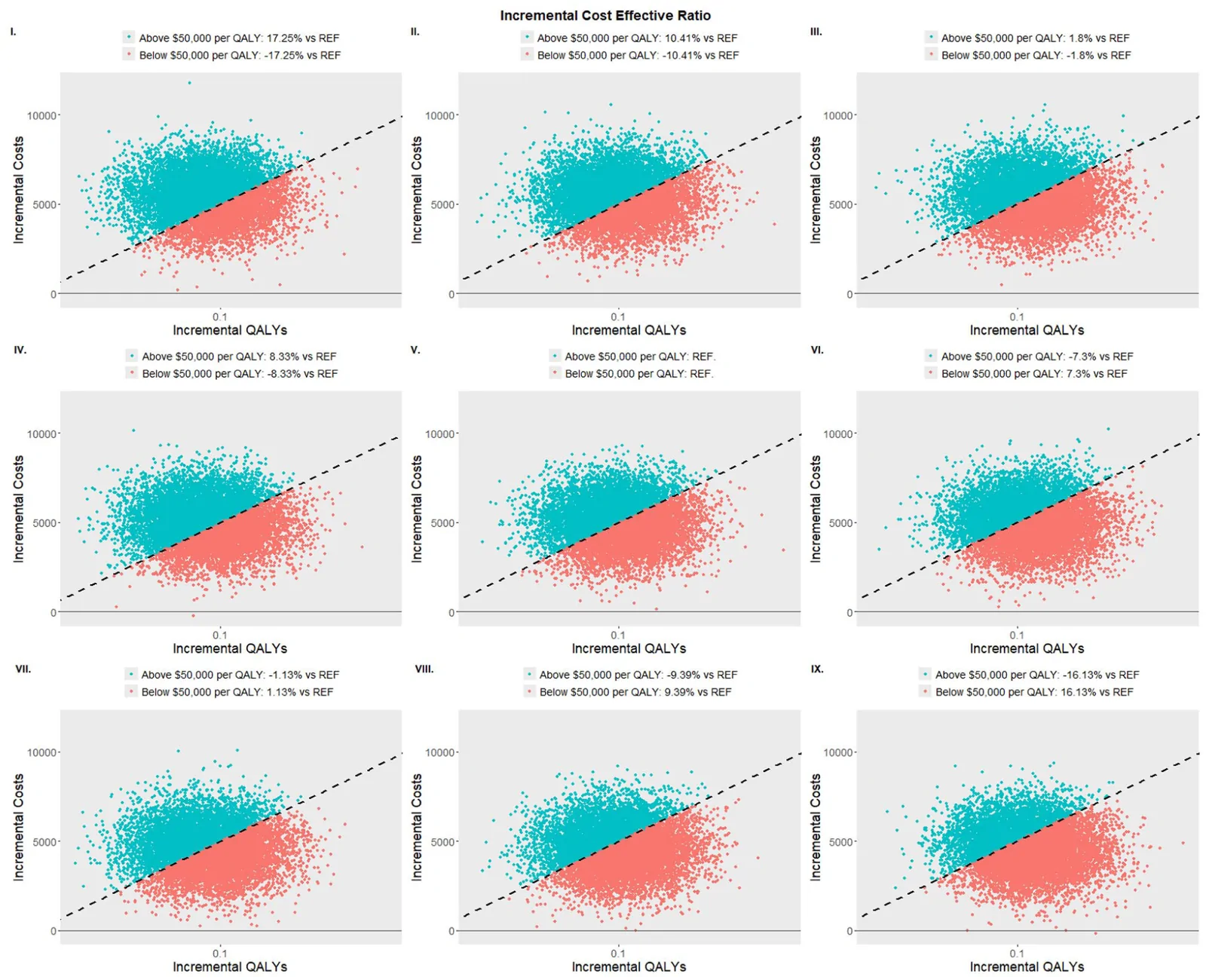
Cost-effectiveness planes under different confounding scenarios.

**Figure 2.**
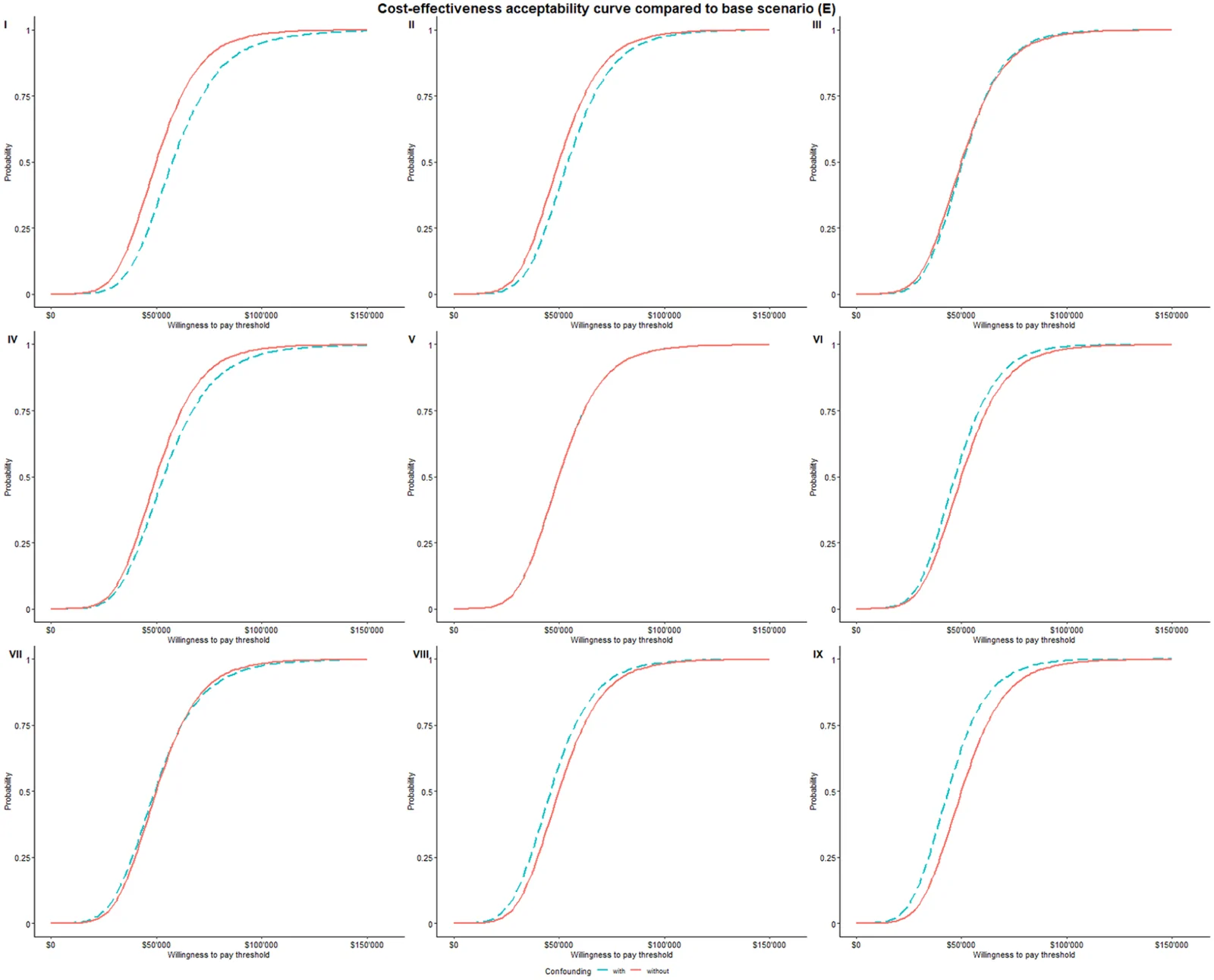
Cost-effectiveness acceptability curves under various confounding scenarios. The full red line present in each panel illustrates the cost-effectiveness acceptability curves (CEACs) one would observe in the absence of confounding. The dashed blue line represents the CEAC one would observe in the presence of the 8 different covariates with the confounding effects that we generated.

The CE planes are provided in Figure 1. Results within the different CE planes illustrate how the radial nature of confounding bias can affect the results of the economic evaluation. For example, in scenario II, where the confounding effect would raise only the ΔC, the new treatment was, at a $50,000 per QALY threshold, deemed to be non–cost-effective in 10.4% more iterations than in the unconfounded scenario V. Alternatively, in scenario VI, where the confounding effect further increased the ΔE, the new treatment was deemed to be cost-effective in 7.3% more iterations than in scenario V. More broadly, the results also illustrated that scenarios in which confounding bias would affect both the incremental cost and effectiveness in opposing manners (scenarios I and IX) had the most important effect on the resulting ICERs. Although specific to our simulations, results in our simulations showed that the combined confounding effect in scenarios I and IX resulted in 17.25% fewer iterations and 16.13% more iterations being considered cost-effective, respectively, compared with the unconfounded scenario V. Similar trends were observed when looking at the resulting CEACs (Figure 2).

## Discussion

The use of real-world data is becoming increasingly widespread in economic evaluation studies.^5,16,19^ Unfortunately, reviews have shown that current practice in the field fails to address the risk of confounding bias expected to occur within such studies.^15–17^ We used simulated datasets in a series of scenarios involving confounders to illustrate the impact of ignoring these confounders within observation economic evaluations. Our findings illustrate that allowing for a confounding effect on cost, on effectiveness, or on both outcomes could result in a change in the key results of the economic evaluation and in the uncertainty around these results. Scenarios in which confounding increases costs and reduces effectiveness (or vice versa) are the most likely to distort the resulting ICERs.

More precisely, we first observe that the presence of confounding factors significantly influenced the distribution of incremental cost and incremental effectiveness within the CE planes (Figure 1) and the resulting CEACs (Figure 2). As expected, these curves show that confounders that shift the resulting ICER upward (by either increasing the incremental costs, decreasing the incremental effectiveness, or both, as in scenarios I, II, and IV) will incorrectly downplay the value of a more effective and more costly intervention, whereas opposite confounding effects (scenarios VI, VIII, and IX) will incorrectly upvalue more effective and more costly interventions.

Where confounders increase or decrease the incremental cost and incremental effectiveness in the same direction (scenarios III and VII), the implications are harder to generalize and may be setting-specific.

Issues we illustrate also raise the underlying question as to when investigators should fear that their conclusions are incorrect and result from incorrectly addressing confounding biases within their analysis. While an important question, its answer likely depends on too many factors, namely, the observed average differential cost and effectiveness (ΔC and ΔE), the study’s effective sample size, and potential confounding variables.^20^ Despite this fact, it remains valuable for investigators facing this risk to reflect on the likelihood that the observed difference in costs (ΔC) and in effectiveness (ΔE) were fully due to an unadjusted confounder. We refer readers to work done by Vanderweele et al^21^ regarding the E-value for risk differences as an example approach while noting that, like for the E-value, this method fails to address how the resulting confounding bias may be due to multiple confounders.

In conclusion, researchers conducting observational economic evaluations must be very mindful of this effect on their conclusions in situations where they expect that overall confounding effects exist. Beyond the greater recognition of risks posed by such biases, further work is needed to better understand which methods most adequately correct these.

## Appendix: Mathematical Details on the Generation of Costs and Effectiveness

Costs were generated according to a Gamma distribution with shape and scale parameters as follows:

ci~Gamma(shape=(μic)2σc2,scale=σc2μic),

with $\sigma_{c}=12,500$ the desired standard deviation for costs and $\mu_{i}^{c}$ the expected mean cost for individual *i*:

$$\mu_{i}^{c}=50,000+5,000t_{j}+\beta_{2}^{c}X_{1i}.$$

The coefficients $\beta_{2}^{c}$ and $\beta_{2}^{e}$ varied between scenarios and were selected as follows:

$$\beta_{2}^{c}=0\mathrm{or}\pm \sigma_{c}\sqrt{\frac{0.1^{2}}{\left(1-0.1^{2}\right)}},\beta_{2,k}^{e}=0\mathrm{or}\pm \sigma_{e}\sqrt{\frac{0.1^{2}}{\left(1-0.1^{2}\right)}},$$

with $\sigma_{e}=0.2$ the residual standard deviation of effectiveness. We can show that this results in a correlation between effectiveness and the covariate of approximately 0.1 conditional on treatment t:

$$\begin{array}{l} Cor(X_{1},e|t)=\frac{Cov(X_{1},e|t)}{\sqrt{Var(X_{1}|t)\times Var(e|t)}}\\ =\frac{Cov(X_{1},0.8+0.1t+\beta_{2}^{e}X_{1}|t)}{\sqrt{Var(X_{1}|t)\times \left[{\left(\beta_{2}^{e}\right)}^{2}Var(X_{1}|t)+\sigma_{e}^{2}\right]}}\\ =\frac{\beta_{2}^{e}\sqrt{Var(X_{1}|t)}}{\sqrt{{\left(\beta_{2}^{e}\right)}^{2}Var(X_{1}|t)+\sigma_{e}^{2}}}. \end{array}$$

If $Var(X_{1}|t)$ were equal to 1, this would be equal to 0.1 exactly. However, since $Var(X_{1})=1$ , and $X_{1}$ and *e* are correlated, the conditional correlation is only approximately 0.1; the true conditional correlation is lower than 0.1 (ie, Cor(X,e |t=1) = 0.0987; Cor(X,e |t=0) = 0.0998). For costs, the same reasoning was used with the additional caveat that costs are not truly a linear function of the covariate and treatment (ie, Cor(X,c |t=1) = 0.0995; Cor(X,c |t=0)=0.0992).
